# Metagenomic insights into respiratory viral signatures in lower respiratory tract infections with and without respiratory failure

**DOI:** 10.3389/fcimb.2025.1637352

**Published:** 2025-09-22

**Authors:** Ning Zhu, Jinying Gao, Ruihong Wu, Shanshan Jia, Xiaohan Guo, Dong Sun, Qingtian Guan

**Affiliations:** ^1^ Bioinformatics Laboratory, Center for Infectious Diseases and Pathogen Biology, The First Hospital of Jilin University, Changchun, China; ^2^ Department of Respiratory Medicine, Center for Infectious Diseases and Pathogen Biology, State Key Laboratory for Diagnosis and Treatment of Severe Zoonotic Infectious Diseases, Key Laboratory for Zoonosis Research of the Ministry of Education, The First Hospital of Jilin University, Changchun, China; ^3^ The Core Facility of the First Hospital of Jilin University, Changchun, China; ^4^ Department of General Surgery, Qilu Hospital of Shandong University, Jinan, China

**Keywords:** lower respiratory tract infections, respiratory failure, microbiome, virome, metagenomic next-generation sequencing

## Abstract

**Objective:**

Lower respiratory tract infections (LRTIs) are a significant cause of morbidity and mortality worldwide, with the respiratory microbiome playing a pivotal role in disease pathogenesis. Comprehensive profiling of the lower respiratory tract virome allows investigation of potential differences between LRTIs and non-LRTIs, helps identify virus-associated taxa linked to pulmonary disease, and provides insights into virome–host interactions involved in respiratory health.

**Methods:**

In this study, we compared viral and bacterial microbiome characteristics of LRTI patients with non-LRTI controls by α-diversity, β-diversity (PCoA, NMDS, ANOSIM), and differential abundance (LEfSe) analyses using metagenomic sequencing of bronchoalveolar lavage fluids, and further performed these comparisons similarly in respiratory failure (RF) patients and non-RF patients in the LRTI group. In addition, virus–bacteria co-occurrence patterns, the correlations between viral and bacterial abundance profiles, and the associations between microbial features and host clinical indicators were assessed using Spearman correlation analysis.

**Results:**

Overall, no significant differences in viral and bacterial α- or β-diversity were detected between LRTI (n=39) and non-LRTI (n=9) groups. However, among LRTI patients with RF (n=5), distinct viral taxonomic signatures were observed, including enrichment of *Phixviricota*, *Malgrandaviricetes*, *Petitvirales*, and *Microviridae* lineages. Despite taxonomic shifts, overall viral diversity remained similar between RF and non-RF subgroups. Bacterial communities showed no notable stratification across clinical categories. Correlation analyses revealed that uncultured human fecal viruses were negatively associated with lymphocyte counts, while Streptococcus-related bacteriophages correlated positively with C-reactive protein (CRP) levels.

**Conclusion:**

The overall composition and diversity of the respiratory microbiome were insufficient to distinguish LRTI from non-LRTI conditions. However, within the LRTI cohort, patients with RF exhibited distinct viral taxonomic profiles compared to non-RF individuals. Additionally, several viral taxa were correlated with host clinical indicators irrespective of clinical subgroup. These findings highlight virome compositional differences associated with RF within LRTI patients, but do not imply causal effects, and warrant further investigation.

## 1 Introduction

Lower respiratory tract infections (LRTIs) are one of the leading causes of death worldwide (World Health Organization, 2024). The most common LRTIs are community-acquired pneumonia (CAP) and hospital-acquired pneumonia (HAP), which differ significantly in their etiology, treatment approaches, and associated mortality rates (GBD 2015 Mortality and Causes of Death Collaborators, 2016; GBD 2016 Lower Respiratory Infections Collaborators, 2018). LRTIs can arise from various viral, bacterial, and fungal pathogens. In addition to common bacterial pathogens such as *Klebsiella pneumoniae*, *Acinetobacter baumannii*, and *Legionella*, viruses are significant causative agents of lower respiratory tract infections, particularly in severe community-acquired respiratory infections (Jain et al., 2015). Based on the limited studies examining the etiology of acute LRTIs in primary care patients, it is evident that acute adult LRTIs in the general community are primarily caused by viral pathogens (Creer et al., 2006). Notable among these are rhinovirus, influenza virus, coronavirus (CoV), *Respiratory syncytial virus* (RSV), *human metapneumovirus* (hMPV), and parainfluenza virus (PiV) (Woodhead et al., 2005; Ieven et al., 2018; Liu et al., 2022). The emergence of the novel severe acute respiratory syndrome coronavirus 2 (SARS-CoV-2) has further underscored the major role of respiratory viruses in LRTIs, highlighting their potential to cause global outbreaks with substantial morbidity and mortality (Wu et al., 2020). However, the pandemic-driven diagnostic and clinical efforts have largely focused on SARS-CoV-2, often overlooking other respiratory viruses. In response, de Campos et al. investigated the viral abundance in pediatric patients with acute respiratory symptoms who tested negative for SARS-CoV-2 (de Campos et al., 2023), which provides valuable insights into the distribution of respiratory viruses and contribute to improved diagnosis and management of pediatric acute respiratory illnesses. Moreover, in some small pediatric studies, respiratory virus testing has been found to reduce antibiotic use (Jennings et al., 2009; Kadmon et al., 2013; Rogers et al., 2015), increase administration of antiviral therapy (Noyola and Demmler, 2000; Kadmon et al., 2013), and reduce the time patients spend in isolation (Iyer et al., 2006; Jennings et al., 2009). The early and accurate identification of viral infections in LRTI patients plays a crucial role in supporting targeted pathogen-specific therapies and facilitating precise clinical decision-making, ultimately promoting improved disease outcomes.

Individuals with immunocompromised states, especially those with pre-existing LRTIs, are at high risk of severe lower respiratory tract complications when exposed to seasonally circulating viruses, compared to healthy individuals. Emerging evidence suggests that persistent infection or colonization of the lower respiratory tract by potential pathogenic bacteria—such as *Haemophilus influenzae* (Cabello et al., 1997; Monsó et al., 1999) and *Pseudomonas aeruginosa* (Hector et al., 2016) observed in diseases like chronic obstructive pulmonary disease (COPD) (Sajjan et al., 2006; Gulraiz et al., 2015) and cystic fibrosis (Chattoraj et al., 2011) respectively can influence subsequent viral infections by upregulating viral entry receptors and modulating inflammatory responses. It is estimated that LRTI due to RSV, a leading cause of acute lung disease, result in 63.8 hospitalizations and 1.04 deaths per 1000 premature children every year worldwide (Stein et al., 2017). Dysbiosis of the respiratory microflora is associated with acute respiratory distress syndrome (ARDS) in critically ill patients, and the ARDS microbiota is characterized by an enrichment of potentially pathogenic respiratory microorganisms, including *Pseudomonas* and *Staphylococcus* spp. (Montassier et al., 2023). However, few researches have focused on characterizing the viral microecology in patients with RF.

Traditional methods for detecting lower respiratory tract pathogens primarily include culture-based techniques, microscopic examination, serological assays, and polymerase chain reaction (PCR) detection. These methods, while widely used, often face challenges such as low sensitivity for fastidious organisms, delayed turnaround times, and the inability to detect non-culturable pathogens or mixed infections. Metagenomic next-generation sequencing (mNGS) offers significant advantages for analyzing bronchoalveolar lavage fluid in diagnosing LRTIs (Simner et al., 2018; Gu et al., 2019). It provides broad-spectrum detection of nearly all known and unknown pathogens, including bacteria, viruses, fungi, and parasites, making it especially useful for identifying hard-to-culture microorganisms. Additionally, mNGS offers high sensitivity and precision, enabling the detection of low-abundance pathogens while distinguishing true pathogens from contaminants (Schlaberg et al., 2017; Blauwkamp et al., 2019). Therefore, this study aims to use mNGS technology to comprehensively profile the lower respiratory tract microecological panorama in patients with LRTIs, with or without RF. Additionally, we performed comparative analyses between LRTI and non-LRTI groups, as well as between LRTI patients with and without RF, to further elucidate the critical role of viruses in respiratory diseases.

## 2 Materials and methods

### 2.1 Patient enrollment

We conducted a prospective cohort study to recruit adult patients with LRTIs or non-LRTIs, with or without concomitant RF, who were hospitalized in the Department of Respiratory Medicine at the First Hospital of Jilin University between January and December 2023. The attending physician combined the patients’ clinical symptoms, laboratory and radiographic results to determine whether they met the criteria for pneumonia infection. Patients in the LRTI group were included based on confirmed or clinically suspected lower respiratory tract infection, defined by pathogen detection (via culture, PCR, or other validated methods) and typical clinical symptoms (e.g., fever, cough, purulent sputum) with radiographic evidence of pulmonary infiltrates. Bronchoalveolar lavage fluid (BALF) samples were collected before or within 48 hours of initiating antimicrobial therapy. The non-LRTI group included patients who showed no clinical, microbiological, or radiographic signs of infection and had clearly defined alternative diagnoses. Exclusion criteria for both groups included incomplete clinical or microbiological data, insufficient BALF volume (<5 mL), poor sample quality (e.g., blood contamination), or use of broad-spectrum antibiotics (>7 days) within one month prior to BAL, except for prophylactic or low-dose regimens. For the non-LRTI group, suspected infection (e.g., unexplained fever or elevated procalcitonin) also led to exclusion. Samples were further excluded post hoc due to sequencing failure or misclassification (e.g., later-confirmed occult infection). Type 1 RF is a partial pressure of oxygen (PaO2) < 60 mmHg with a normal or decreased partial pressure of carbon dioxide (PaCO2). Patients with congenital immunodeficiency disorders, those on long-term immunosuppressive therapy, or those who received corticosteroids or other hormonal treatments within 48 hours prior to enrollment were excluded. For all subjects included in the study, specimens were discarded if consent was not ultimately obtained from the patient or their proxy.

### 2.2 Clinical sample collection, whole-genome sequencing and data quality control

BALF from a total of 48 patients was collected from the Department of Respiratory Medicine of the First Hospital of Jilin University. Among them, 39 patients were clinically diagnosed with LRTI, while 9 patients served as non-LRTI control, who did not meet the clinical or microbiological criteria for LRTI but underwent bronchoscopy due to other respiratory indications. Within the LRTI group, a further classification was performed based on clinical severity: 5 patients presented with RF, and the remaining 34 patients did not develop RF. Genomic DNA was extracted from samples using QIAamp^®^ UCP Pathogen DNA Kit (Qiagen) following the manufacturer’s instructions. DNA concentration was quantified using a Qubit fluorometer (Thermo Fisher Scientific, USA). Human DNA was removed using Benzonase (Qiagen) and Tween20 (Sigma). For library preparation, genomic DNA was enzymatically fragmented, with an average insert size of approximately 300 bp. The quality and size distribution of libraries were assessed using a Bioanalyzer (Agilent Technologies, USA). Libraries were then constructed using Ovation Ultralow System V2 (NuGEN, CA, USA) and sequenced on Illumina Nextseq 550 (Single-End 75bp) platform. The raw sequencing data was first subjected to quality control using FastQC v0.12.1 (Vashishtha et al., 2022) and was subsequently processed to remove reads containing adapters, phix, and trimming of bases with quality scores below 30. Above obtained cleaned reads were then aligned to the human genome hg19 with default parameters, and the unmatched reads were retained for further analysis, all of which were performed using BBMap v39.06 (Bushnell, 2014).

### 2.3 Genome assembly and microecological species composition

To gain a comprehensive understanding of respiratory microbiological characteristics, we employed two analytical approaches: an exact k-mer matching metagenomic pipeline Kraken2 v2.1.2 (Wood et al., 2019) and Bracken v2.9 (Lu et al., 2017) (with a 8 Gb MiniKraken database constructed on 3 April 2018) for rapid taxonomic classification and a metagenome-assembled genomes (MAGs) pipeline (MetaWRAP v1.3.2 (Uritskiy et al., 2018)) for bacterial assembly. Bacterial species with a relative abundance greater than 0.01% were included in the analysis. Viral taxonomic classification of contigs was performed using BLASTn 2.15.0+ (Camacho et al., 2009) to query the NCBI nucleotide database. The identity, mapped length and e-value cutoff was set at 80%, 150bp and 1e-5 respectively to maintain high sensitivity at a low false-positive rate. The taxonomic lineage of the top blast hit for each contig was determined, and those classified under the kingdom “Viruses” were identified as potential viral hits. Meanwhile, the decontam R package (Davis et al., 2018) was applied to identify and remove potential contaminants from viral taxonomic profiles. As negative control samples were not available, the “frequency” method was employed, using total sequencing reads per sample as a proxy for DNA concentration. Viral features classified as contaminants (*p* < 0.1) were excluded from subsequent analyses. Moreover, clean reads are mapped to the DNA sequences of the core dataset from the Virulence Factor Database (VFDB) via BBMap v 39.06 (Bushnell, 2014) (with default parameter). The output includes ‘.sam’ file and ‘.rpkm’ file, which contains the mapping details for each read and provides the total read count for each sample. The fpkm values for each virulence factor are subsequently compared differentially between the two groups.

### 2.4 Data representation and statistical analyses

To explore the relationship between viruses and bacteria, the co-occurrence between viruses and respiratory bacteria was assessed using Fisher’s exact test, and Spearman correlation tests between viral abundance and bacterial abundance were conducted, followed by Benjamini–Hochberg (BH) correction. Here, viral abundance was uniformly quantified using Log10 (Reads Per Million (RPM)), RPM = Mapped reads*10^6^/Total reads, and bacterial abundance is relative abundance. These two analyses are visualized as heatmaps and Chord diagram, respectively. A virus or bacterium is excluded from the representation if it coexists with all bacteria in fewer than five samples or if it coexists with all viruses in fewer than five samples. Only virus-bacteria correlations with a coefficient greater than 0.6 were included in the Chord diagram. Spearman analysis was performed to examine the associations between the presence and abundance of each virus and relevant clinical indications; no multiple testing correction was applied. The microbial composition of the sample was presented by the distribution of individual bacteria and viruses. The alpha diversity index was used to assess the taxonomic diversity of each sample, and the Wilcoxon rank-sum test was applied to determine differences between the two groups. The compositional variation of microbial communities across samples was assessed using beta diversity analysis based on Bray-Curtis distance metrics. The results were visualized through Principal Coordinate Analysis (PCoA), Non-Metric Multidimensional Scaling (NMDS), Analysis of Similarities (ANOSIM) and a dendrogram generated from hierarchical clustering. LEfSe (Linear Discriminant Analysis Effect Size) analysis was performed using the microeco R package. The Kruskal–Wallis test was applied without FDR correction for the main LEfSe workflow, and a linear discriminant analysis (LDA) score threshold of 3.0 was used to retain features with strong discriminatory power. Additionally, Welch’s t-test and Kruskal–Wallis test with Benjamini–Hochberg correction (wi.eBH and kw.eBH) from the ALDEx2 framework were applied to assist significance assessment and enhance the robustness of differential feature identification. The analyses were visualized using vegan, ggplot2, microeco, pheatmap, Hmisc, and igraph packages in R studio 4.3.3.

## 3 Results

### 3.1 Clinical characteristics

A total of 48 patients hospitalized in the Department of Respiratory Medicine at the First Hospital of Jilin University between January and September 2023 were enrolled in this study (**Tables 1**–**3**; **Supplementary Table 1**). The median age was 55 years, and 54% of the patients were male. Most individuals had underlying comorbidities, including 16 with other pulmonary diseases (e.g., COPD, bronchiectasis, and lung cancer), 10 with diabetes, and 7 with RF or hypoxemia. Among the enrolled patients, 39 were diagnosed with LRTIs, of whom 5 had concomitant RF. The remaining 9 patients without LRTI served as the non-LRTI control group. Laboratory tests identified various bacteria including *Mycobacterium tuberculosis*, *Pseudomonas aeruginosa*, *Staphylococcus aureus*, *Pneumocystis japonicum*, *Klebsiella pneumoniae*, *Acinetobacter baumannii*, *Haemophilus influenzae*, and *Streptococcus pneumoniae*, and a number of fungi and viruses, more commonly *Human gammaherpesvirus 4* (HHV-4) and *Cytomegalovirus* (CMV), were also detected. The hospitalization duration for these patients ranged from 3 to 22 days, with a median of 9.45 days, and no mortality events were reported. Baseline demographic and clinical characteristics of the study participants, including age, sex, diagnosis, comorbidities, and laboratory parameters, are summarized in **Supplementary Table 1**.

**Table 1:** Summary of study participants.

Characteristic	Value (n=48)
Gender	
Male	26 (54%)
Female	22 (46%)
Median age, years	55 (33-72)
Age by sex, years	
Male	58 (34-72)
Female	54 (33-72)
Primary disease	
LRTI	39 (81%)
non-LRTI	9 (19%)
Complications	
Other respiratory diseases	16 (33%)
RF	7 (15%)
Hypoxemia	7 (15%)
Diabetes	10 (21%)
Hypertension	13 (27%)
Median duration of hospital admission; Days	9.45 (3-22)
Outcome at hospital discharge	
Stable, discharged home	30
Specialized hospital treatment after discharge	18

**Table 2:** Primary diseases and comorbidities.

Category	Subcategory	n (%)
Primary diseases		
LRTI	Total (see **Table 3** for breakdown)	39(81%)
Non-LRTI	Total	9 (19%)
	Interstitial lung disease	4 (8%)
	Pulmonary alveolar proteinosis	1 (2%)
	Vasculitis	1 (2%)
	Organizing pneumonia	1 (2%)
	Lung cancer	1 (2%)
	Others	2 (4%)
Comorbidities		
Other respiratory diseases	COPD	5 (10%)
	Asthma	2 (4%)
	Bronchiectasis	4 (8%)
	Pulmonary nodules	3 (6%)
	Pleural effusion	4 (8%)
	Lung caner	4 (8%)
Systemic diseases	Hypertension	13 (27%)
	Diabetes	10 (21%)
Critical illness	RF	7 (15%)
	Hypoxemia	7 (15%)

**Table 3:** Pathogen distribution in LRTI group (n=39).

Pathogen category	n (%)	Specific pathogens (n)
Bacteria	29 (74%)	*Mycobacterium tuberculosis* (9)
		*Staphylococcus aureus* (5) *Klebsiella pneumoniae* (4)
		Others (6)
Fungi	14 (29%)	*Candida* spp. (7)
		*Aspergillus* spp. (6)
		*Pneumocystis jirovecii* (5)
Virus	8 (17%)	HHV-4 (8)
		CMV (4)
No pathogen identified	9 (19%)	

LRTI, Lower Respiratory Tract Infection; RF, Respiratory Failure; HHV-4, *Human gammaherpesvirus 4*; CMV, *Cytomegalovirus*; COPD, Chronic Obstructive Pulmonary Disease.

### 3.2 The distribution of the lower respiratory tract microbiome

**Figure 1:**
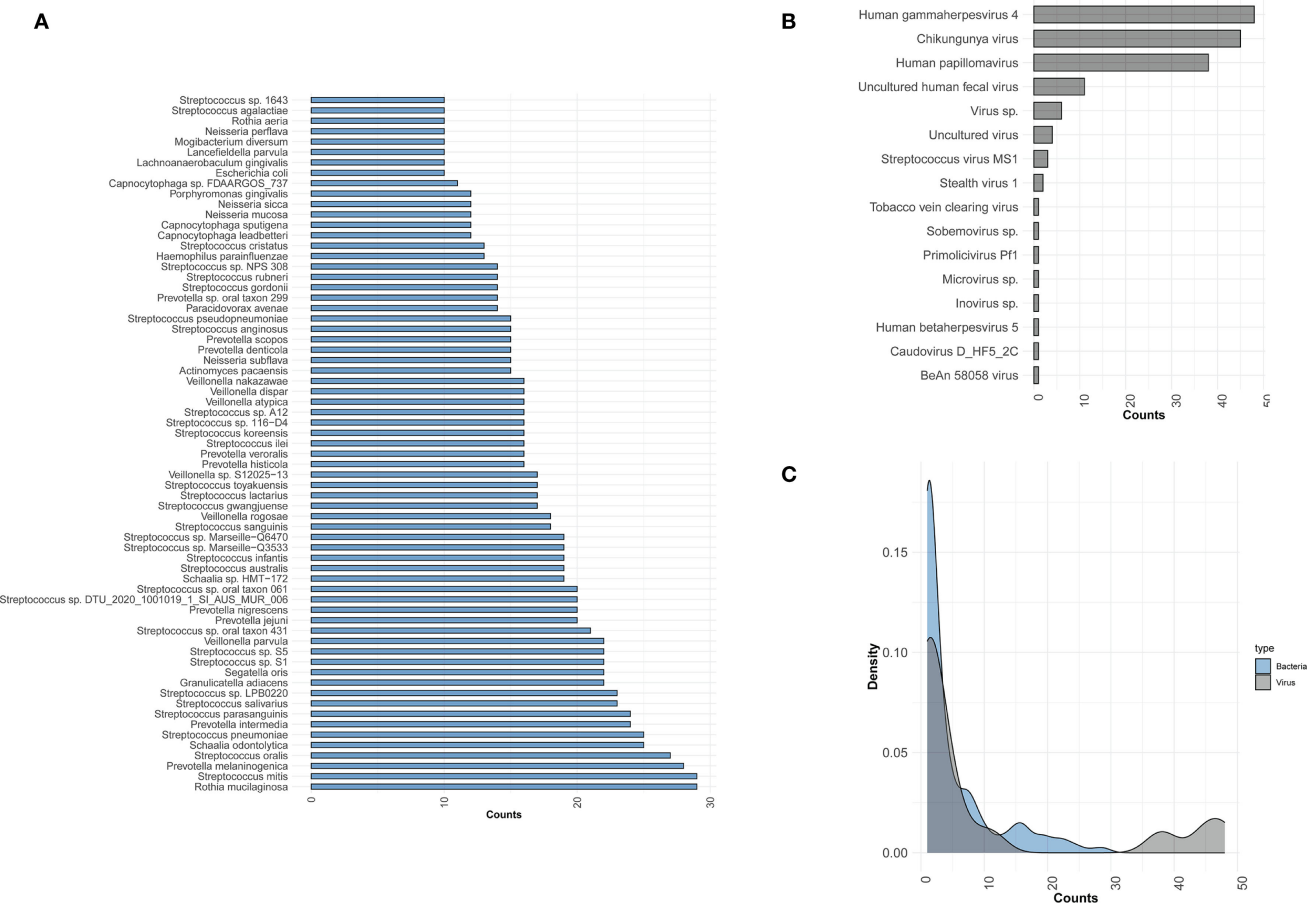
Distribution of bacteria and viruses detected by mNGS. **(A)** Bar plot showing the bacterial species distribution across samples; only species detected in ≥10 samples are included. **(B)** The distribution of viruses across all samples. **(C)** The density distribution of samples for viruses and bacteria. The x-axis represents the counts of samples detected for bacteria or viruses, while the y-axis represents the density.

To identify potential pathogens across all samples, we performed second-generation sequencing and generated pathogen profiles for each sample. A total of 16 viruses and 410 bacterial species were identified across 48 samples. Of these, 6 samples were excluded based on bacterial abundance criteria, leaving no detectable bacterial species in those samples. To streamline the analysis, we highlighted the distribution of bacterial species present in 10 or more samples, along with all detected viruses, and analyzed their count density distributions (**Figures 1A–C**). The top 5 bacteria species within all the samples were *Rothia mucilaginosa* (*R. mucilaginosa*), *Streptococcus mitis* (*S. mitis*), *Prevotella melaninogenica* (*P. melaninogenica*), *Streptococcus oralis* (*S. oralis*), *Schaalia odontolytica* (*S. odontolytica*). Viral pathogens were detected in all of patient samples, and at least two viruses were identified in each sample. The most frequently detected virus was *Human gammaherpesvirus 4* (HHV-4), present in all samples (48/48), followed by *Chikungunya virus* (CHIKV, 45/48), and *Human papillomavirus* (HPV, 38/48). In this study, HPV refers to both *Human papillomavirus 16* and *Human papillomavirus 18*.

### 3.3 The association between viruses and the microbial community

**Figure 2:**
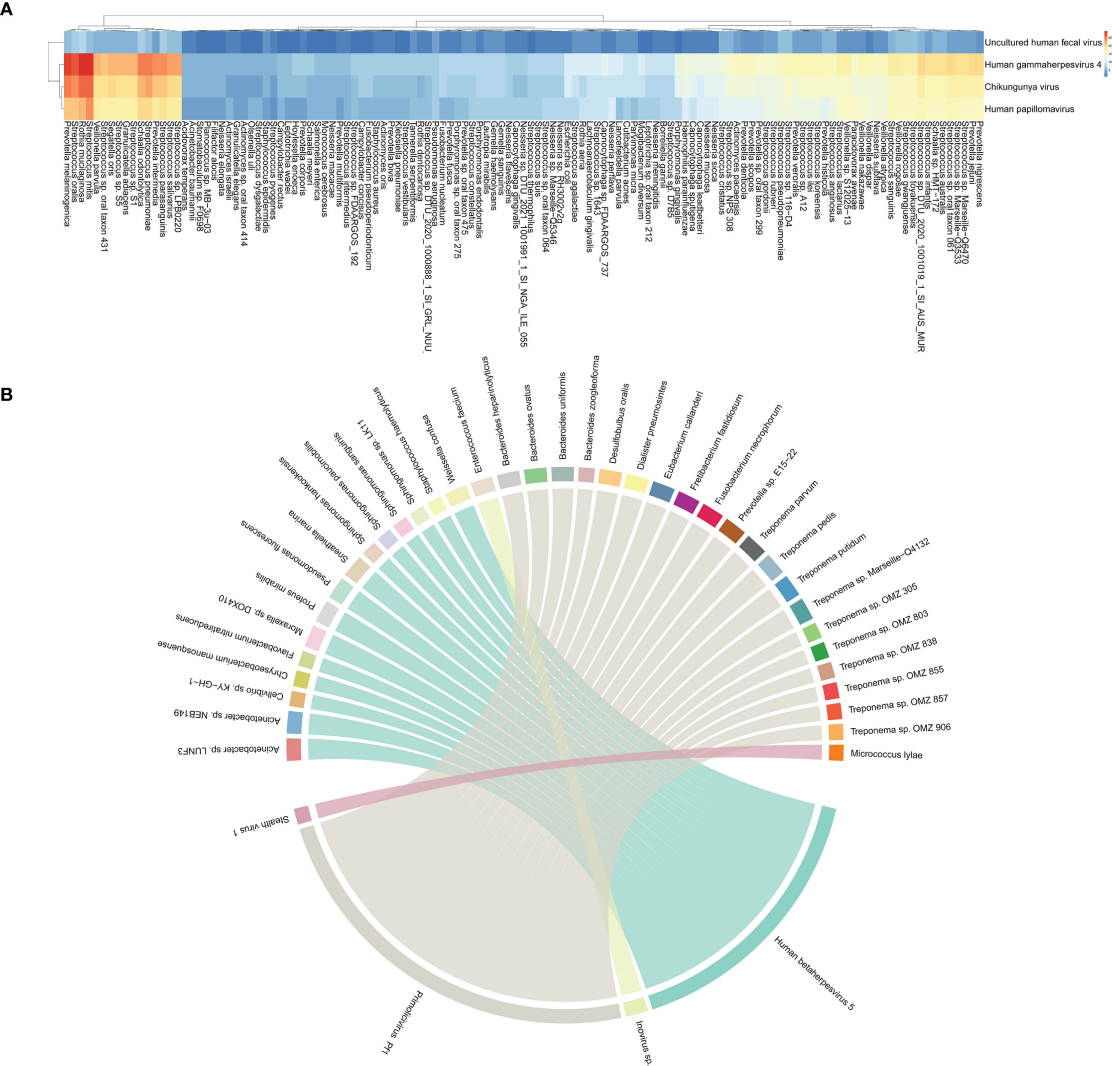
Interconnection of bacteria and viruses. **(A)** Heatmap of co-occurrence matrix between 4 viruses and 125 bacteria, reflecting the frequency of two microorganisms co-occurring. **(B)** The chord diagram shows virus–bacterium pairs with Spearman correlation coefficients greater than 0.6. Each segment on the outer circle represents a viral or bacterial species, color-coded to distinguish nodes. The width of the connecting ribbons is proportional to the strength of the correlation.

To assess the ecological relationships among microbes in these samples, we constructed a microbial co-occurrence network (CoN) as described previously. The specific viruses that tend to co-occur with distinct bacteria were distinguishable within each subject (**Figure 2A**). A heatmap was constructed based on the co-occurrence matrices, with blue and red indicating lower and higher frequencies of co-occurrence, respectively. The quantified co-occurrence heatmap showed the tightness in HHV-4, CHIKV, HPV with *P. melaninogenica*, *R. mucilaginosa*, *S. mitis*, *S. oralis*, and *S. pneumoniae*. However, this is a randomly occurring result and does not indicate that this co-occurrence is statistically significant. Consistent with our first result, viral infection frequently has been associated with carriage of common pathogens. To further explore the relationship between viruses and bacteria, we conducted a correlation analysis based on their abundance profiles (**Figure 2B**), where each ribbon represents a significant correlation between a virus and a bacterium. The width of each ribbon reflects the strength of the correlation, allowing the identification of bacterial taxa most strongly associated with each virus. Based on the bacterial and viral abundance correlation results we obtained (**Supplementary Table 2**), the abundances of *Human betaherpesvirus 5* and *Primolicivirus Pf1* were positively correlated with the abundances of various bacteria. *Human betaherpesvirus 5* (HHV-5), a beta-herpesvirus that is highly prevalent worldwide, has the ability to establish lifelong latency in its host following primary infection. The abundance of HHV-5 was found to exhibit a perfect positive correlation (correlation coefficient r = 1) with the abundances of several bacterial taxa, including *Acinetobacter* sp. LUNF3, *Acinetobacter* sp. NEB149, *Moraxella* sp. DOX410, *Proteus mirabilis*, *Pseudomonas fluorescens*, *Sneathiella marina*, *Weissella confusa*. *Primolicivirus Pf1* is a filamentous bacteriophage belonging to the *Inoviridae* family, known to infect *Pseudomonas aeruginosa* and persist as an episome or integrated into the bacterial chromosome. In our study, Pf1 abundance exhibited a strong positive correlation (correlation coefficient r > 0.6) with several members of the *Treponema* genus, including *Treponema vincentii* and *Treponema* strains OMZ 305, OMZ 803, OMZ 838, OMZ 855, OMZ 857, and OMZ 906. Although *Treponema* species are not known natural hosts of Pf1, this association may reflect co-occurrence patterns within shared ecological niches, such as mucosal surfaces or inflamed tissue environments. These correlations suggest that phage-bacterium interactions, even among noncanonical host relationships, may shape microbial community dynamics and warrant further investigation to elucidate their functional implications.

### 3.4 Altered virome in the lower respiratory tract associates with clinical indicators

**Figure 3:**
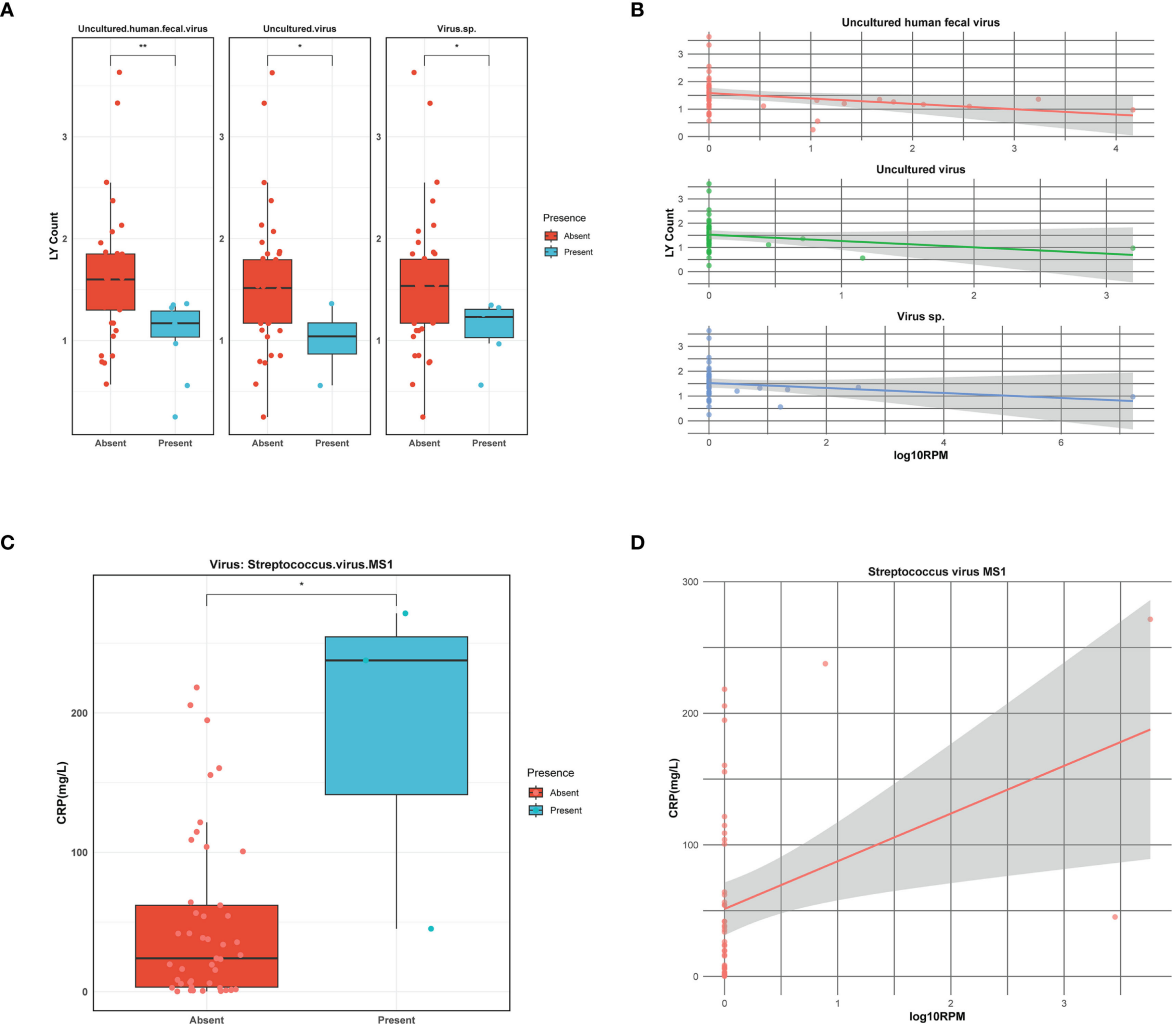
Correlation of viruses with clinical indicators. **(A)** Boxplot illustrating lymphocyte (LY) count in the presence or absence of viruses. Asterisks denote statistically significant differences given by Wilcoxon rank-sum test (*p < 0.05, **0.001≤*p*<0.01). **(B)** The linear relationship between viral abundance and lymphocyte (LY) count. Viral abundance is expressed as log10RPM. **(C)** Boxplot illustrating C-reactive protein (CRP) level in the presence or absence of viruses. Asterisks denote statistically significant differences given by Wilcoxon rank-sum test (*p < 0.05). **(D)** The linear relationship between viral abundance and C-reactive protein (CRP) level. Viral abundance is expressed as log10RPM.

Viral infections are often associated with changes in certain inflammatory markers. To explore the relationship between viral infections and clinical indicators, a Spearman analysis was conducted between viral presence, their abundance and some clinical markers, including white blood cell (WBC), neutrophilic granulocyte (NE), lymphocyte (LY), c-reactive protein (CRP), erythrocyte sedimentation rate (ESR), procalcitonin (PCT), and the length of hospitalization across all samples. We observed that *uncultured human fecal virus*, *uncultured virus*, *virus.sp*. have an impact on LY counts in serum, with higher viral abundance correlating with lower LY counts (**Figures 3A, B**). Additionally, *Streptococcus virus MS1* directly influences CRP levels, where an increase in viral abundance is associated with a corresponding rise in CRP levels (**Figures 3C, D**). The findings suggest that different viral infections exert distinct effects on the host, and the associated laboratory markers exhibit a certain correlation with viral abundance.

### 3.5 Discriminant analysis of patients with LRTI

**Figure 4:**
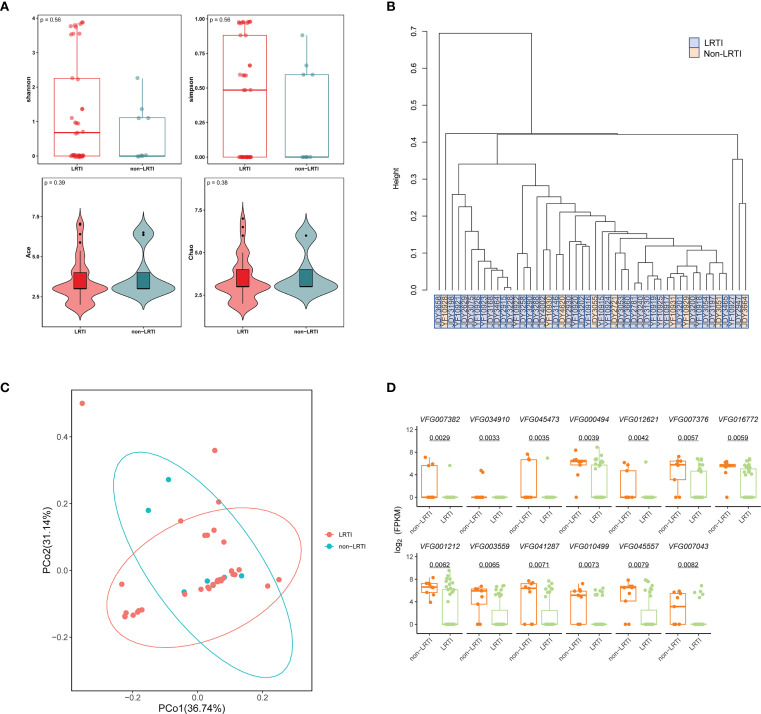
Comparison of respiratory virome and virulence factors in LRTI and non-LRTI groups. **(A)** Alpha diversity of the respiratory virus between patients with LRTI and non-LRTI. **(B)** Hierarchical clustering of LRTI and non-LRTI groups based on Bray-Curtis distances of viral composition. **(C)** Principal Coordinate Analysis (PCoA) with Bray-Curtis distance and Classical Multidimensional Scaling (CMDS) to test for differences in communities between groups. Each color represents one of the analysis groups specified in the legend. **(D)** Box plots of comparison of logarithmic values of the virulence factor FPKM in LRTI and non-LRTI groups. The top of each box plot is the corresponding virulence factor.

To investigate lower respiratory tract virus signatures in LRTI and non-LRTI individuals, we assessed the diversity of virus in two cohorts. Alpha diversity analysis showed no significant differences in Shannon, Simpson, ACE, and Chao indexes (*p* = 0.56, *p* = 0.56, *p* = 0.39, *p* = 0.38) between LRTI and non-LRTI groups (**Figure 4A**). Furthermore, the hierarchical clustering based on the Bray-Curtis distance of the composition of virome did not clearly separate the two cohorts (**Figure 4B**). Similarly, PCoA analysis didn’t capture significant separation between the LRTI and non-LRTI cohorts (**Figure 4C**). To determine whether there are any significant different taxa in respiratory virome between the two groups, LEfSe analysis was conducted, and results did not show any significant differences.

Similarly, in order to characterize the lower respiratory tract bacterial communities, we compared metagenomic data between LRTI and non-LRTI groups. Alpha diversity analysis revealed no significant differences in richness or diversity between the two groups (**Supplementary Figure 1A**). Additionally, beta diversity analyses, including PCoA and NMDS based on Bray-Curtis distances, as well as ANOSIM, showed no discernible differences in the respiratory bacterial microbiota between the two groups (**Supplementary Figures 1B–D**). Further LEfSe analysis did not identify any bacterial taxa with significant differential abundance between the two groups. Collectively, these findings suggest that the composition and diversity of the respiratory bacterial community or viral communities, when considered independently, may not be sufficient to reliably distinguish lower respiratory tract infections (LRTIs) from non-LRTI conditions.

In addition, we matched virulence factors in all samples from VFDB, and 13 virulence factors were significantly different between the two groups with *p*<0.01 (**Figure 4D**), including Effector delivery system, Adherence, Motility, Exotoxin, Regulation, Nutritional/Metabolic factor, Immune modulation, and the median of the fpkm levels of these virulence factors was higher in the non-LRTI group compared to the LRTI group.

### 3.6 Discriminant analysis of LRTI patients combined with RF

**Figure 5:**
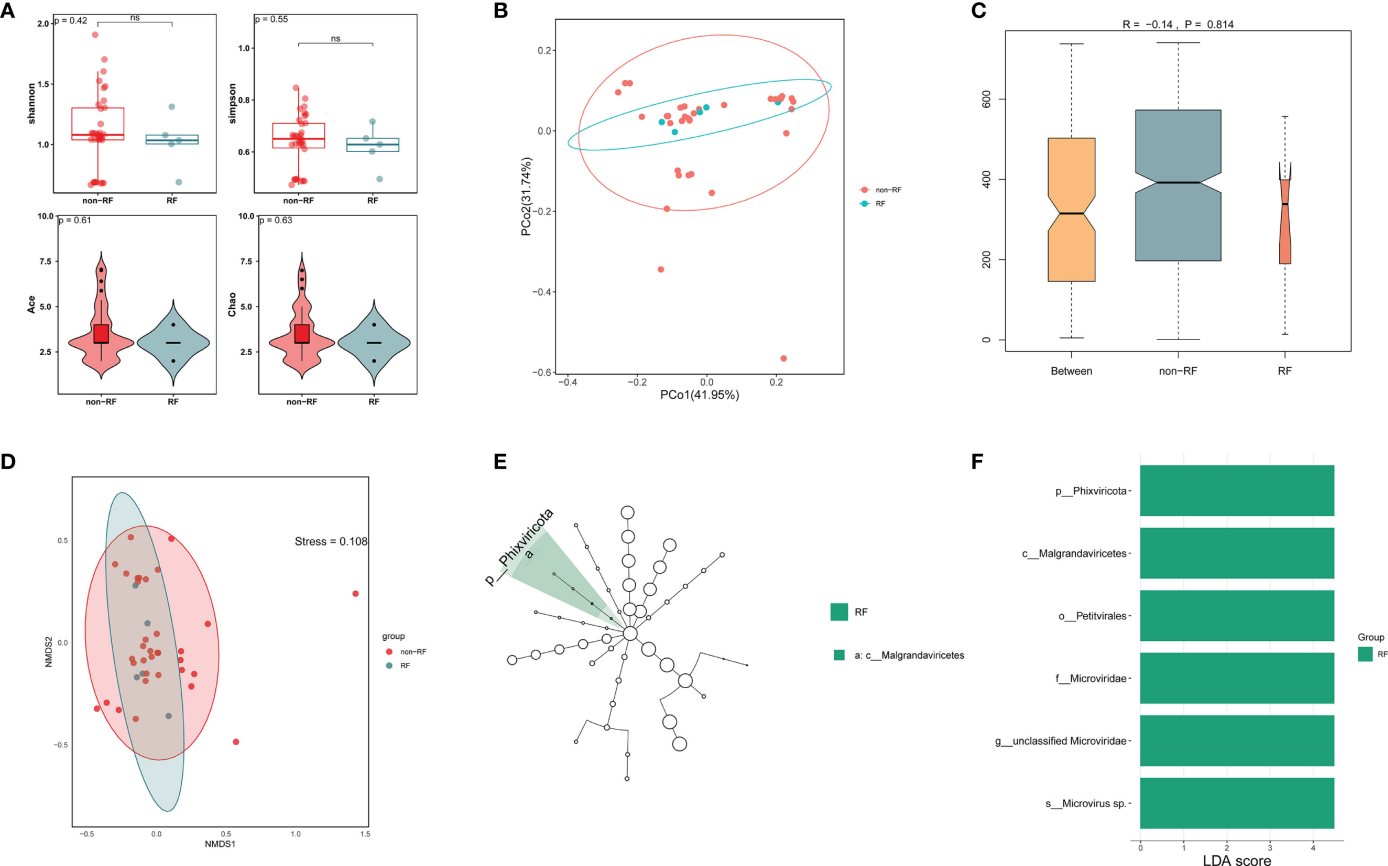
Comparative virome analysis in LRTI with RF and without RF groups. **(A)** Alpha diversity of the respiratory virus between LRTI with RF and without RF groups. **(B)** Principal Coordinate Analysis (PCoA) with Bray-Curtis distance and Classical Multidimensional Scaling (CMDS) to test for differences in viral communities. Each color represents one of the analysis groups specified in the legend. **(C)** Analysis of Similarities (ANOSIM) test with Bray-Curtis distance to test for differences in viral communities between two groups. **(D)** Non-Metric Multidimensional Scaling (NMDS) analysis with Bray-Curtis distance shows differences in viral communities between two groups. **(E, F)** LEfSe analysis identifies significantly enriched viral taxa (LDA score >3, *p* < 0.05) in LRTI with RF versus LRTI without RF, with bar lengths representing the effect size of each taxon's contribution to group differences.

To investigate potential viral differences between LRTI patients with and without RF, we conducted alpha-diversity analysis along with PCoA, NMDS, and ANOSIM to assess variations between the two groups. The alpha-diversity analysis showed no significant differences in richness and diversity between LRTIs with and without RF groups (**Figure 5A**). PCoA and NMDS analyses of Bray-Curtis distance (**Figures 5B, D**) showed that the virome in the LRTIs with RF group was not apart from that in the LRTIs without RF group (stress=0.108). According to the ANOSIM test, there were no significant differences in viral microbial composition between the two groups, with comparable levels of similarity between and within groups (**Figure 5C**). However, LEfSe analysis was further performed to determine specific taxonomic groups with abundance changes, which revealed that *Phixviricota*, *Malgrandaviricetes*, *Petitvirales*, *Microviridae*, unclassified *Microviridae*, and *Microvirus* sp. were significantly enriched in the LRTIs with RF group compared to the LRTIs without RF group (**Figures 5E, F**). In contrast, no significantly differential taxa were identified in the LRTIs without RF group compared to the LRTIs with RF group. These results indicate that some significantly unique viruses are enriched in the lower respiratory tract of LRTI patients with RF compared to those without RF. Moreover, we performed a differential analysis of bacterial microbiota between subgroups, bacterial communities showed no distinct α/β-diversity patterns (**Supplementary Figure 2**).

## 4 Discussion

In this study, we investigated LRTIs with or without RF using mNGS to detect the lower respiratory tract microbiota. A total of 48 samples were ultimately included in this study, which is the first prospective study to date to characterize the lower respiratory virome in adult patients with LRTI or LRTI and RF.

This study began by determining the overall composition of bacteria and viruses in all samples, where the detection rate of viruses was relatively low, with a total of 16 viruses present in all samples, with at least two viruses being detected in each sample. The top three viruses were HHV-4, CHIKV, and HPV, with HHV-4 topping the list of all viruses in terms of detection rate, a result that is consistent with a previous study in which HHV-4 was detected in 5.5% of samples (Jin et al., 2022). The interplay between viruses and bacteria in the development of respiratory infections has been thoroughly documented in the literature. Studies have shown that virus-bacterium and virus-virus coinfections are common in both children and adults with CAP (Honkinen et al., 2012; Jain et al., 2015; Wei et al., 2015). The most well-known virus-bacteria interaction is the synergistic relationship between influenza viruses and *S. pneumoniae* (McCullers, 2006). Also, Human rhinovirus (HRV), Influenza virus and *S. pneumoniae* (McCullers and Rehg, 2002; Wiertsema et al., 2011), HRV and *S. aureus* (Wang et al., 2009), and hMPV and *S. pneumoniae* (Verkaik et al., 2011) interactions have been described in the literature. In our study, certain bacteria and viruses were identified as coexisting and interconnected, although these viruses were not the primary pathogens causing pneumonia. Not surprisingly, the top three viruses detected most frequently in the samples coexisted with the top five bacteria. In addition, we found that the abundances of certain viruses, including HCMV, *Inovirus*, and *Primolicivirus*, were strongly correlated with those of certain bacteria (r = 1), such as *Acinetobacter* sp. LUNF3, *Enterococcus faecium*, *P. fluorescens* (**Supplementary Table 1**). Although viral presence alters the respiratory environment and influences microbial community structure, the underlying mechanisms remain unclear in existing studies.

In individuals with viral infections, serological parameters are often altered. For example, the researchers found that the presence of viruses other than adenovirus in BALF from asymptomatic children undergoing elective surgery was associated with significantly higher neutrophil counts but not with macrophage, lymphocyte, or eosinophil counts, which could mean that a low viral load triggers only a mild inflammatory response and does not lead to respiratory symptoms (Thavagnanam et al., 2010). In our study, a correlation analysis between the presence or absence of the virus and the abundance of the virus and the serological indexes showed some positive results (**Figure 3**). Specifically, *uncultured human fecal virus*, *uncultured virus*, *virus.sp* had a significant negative linear correlation with LY counts, but these three types of viruses are all unclassified viruses and are not documented by the literature, so it can be speculated that the above viruses cause a decrease in LY counts in the early stages of infection. Additionally, we found that streptococcal virus MS1 had a significant positive correlation with CRP, and the higher the viral abundance, the higher the CRP level. CRP contributes to host defense by providing protection against *S. pneumoniae* infection, as demonstrated in murine models of pneumococcal disease (Ngwa and Agrawal, 2019). *Streptococcus* viruses, a class of bacteriophages that specifically target bacteria of the *Streptococcus* genus, also act as natural antibacterial agents. Interestingly, CRP functions as a broad-spectrum antibacterial molecule, and this defensive role partially mirrors the antibacterial activity of *Streptococcus*-targeting phages. This functional similarity may help explain the observed positive correlation between elevated CRP levels and the abundance of *Streptococcus* phages. However, it is important to note that our study is cross-sectional and relies on correlation-based analyses of metagenomic data, without longitudinal follow-up or experimental validation. As such, the observed virus-host or virus–bacterium associations should be interpreted as exploratory rather than indicative of direct biological interactions. Future studies incorporating temporal data or functional assays will be necessary to confirm the nature and significance of these associations.

It is well established that respiratory viruses, particularly influenza viruses, increase susceptibility to secondary bacterial infections (McCullers, 2006). Numerous mechanisms, including disruption of physical and immune barriers (Herold et al., 2008) as well as alterations in the microenvironment, have been implicated in facilitating such infections. In contrast, our understanding of how bacteria influence the host’s response to subsequent viral infections remains limited. There is no doubt that lung diseases influence the lower respiratory microbial community. During lung infections, viral species diversity tends to decrease, and when accompanied by RF, the overall taxonomic composition of viruses undergoes notable changes. Notably, we observed significant enrichment of the *Phixviricota*, *Malgrandaviricetes*, *Petitvirales*, *Microviridae*, unclassified *Microviridae*, and *Microvirus* sp. in individuals with LRTI and RF (**Figure 5F**). However, the detected viruses were not identified as pathogenic agents, and their viral loads were relatively low. There is a significant lack of studies investigating the virome in RF cases that are not directly attributed to viral infections. Further investigation through large-scale cohort studies is needed to validate and explore these findings in greater detail. Interestingly, bacterial communities showed no distinct α/β-diversity patterns and differential taxonomic groups via PCoA, NMDS, ANOSIM, or LEfSe analyses between LRTI and non-LRTI groups, and there was no difference in bacterial species diversity between the LTRI with RF and the LRTI without RF groups. Furthermore, the median FPKM values of virulence factors were significantly higher in the non-LRTI group compared to the LRTI group.

Virulence factors are effectors that enable pathogens to colonize ecological niches in the host, immune evasion, suppression of host immune responses, movement in and out of cells, and acquisition of nutrients from the host, and are important in the process of disease development. For example, *M. tuberculosis* induces cellular necrosis through virulence factors such as EsxA, CpnT and PDIM, thereby facilitating the transmission of *M. tuberculosis* (Chandra et al., 2022). These findings suggest that viral diversity may play a protective or regulatory role in maintaining microbial balance in the respiratory tract. The significantly elevated abundance of virulence factors observed in the non-LRTI group can be attributed to several plausible factors. First, in non-infected individuals, the respiratory microbiome tends to be more diverse and compositionally balanced. Many commensal or environmental bacteria in such communities may harbor genes associated with virulence—such as motility (e.g., VFG007382, VFG007376, VFG016772), effector delivery systems (e.g., VFG034910, VFG003559, VFG000494), or metabolic factors—but these genes often exist in a low-activity or latent state and do not necessarily indicate pathogenic behavior. Second, the presence of environmental or non-pathogenic microorganisms in non-LRTI samples may contribute to an apparent increase in the abundance of virulence-associated genes, simply due to gene presence rather than functional expression. In contrast, LRTI samples may exhibit microbial dysbiosis dominated by one or a few pathogenic species, potentially leading to a narrower set of virulence strategies and reduced diversity in detectable virulence genes. Therefore, the higher FPKM values observed in the non-LRTI group likely reflect ecological diversity and broader gene content rather than active virulence or infection-related gene upregulation.

When viral infections occur there is a strong association with bacteria, e.g., viral infections alter host epithelial cell defenses making epithelial cells more susceptible to bacterial colonization (Bogaert et al., 2004; Vareille et al., 2011); viral infections also trigger a series of pro-inflammatory responses leading to the up-regulation of adhesion proteins in a range of cells, including epithelial cells (Jiang et al., 1999; Avadhanula et al., 2006), and increased expression of these adhesion proteins may promote adhesion of certain bacteria, such as *S. pneumoniae* and *H. influenzae* (Ishizuka et al., 2003; Avadhanula et al., 2006; Wang et al., 2009). Similarly, when bacterial infections are interlinked with viruses, the composition of the bacterial microbiome may be associated with altered risk (or outcome) of viral infections, and this co-pathogenesis may be due to the following (Bellinghausen et al., 2016) 1) e.g., impaired immune function or increased exposure that make patients more susceptible to infections with both pathogens; 2) increased risk of viral infections, e.g., up-regulation of the viral entry receptor; and 3) prolonged exposure to pathogenic bacteria in patients with altered responses to viruses, such as synergistic effects on inflammation or tissue damage. Mechanistically, however, the question of how bacterial colonization of the lungs or chronic bacterial infection affects susceptibility to (and outcome of) subsequent viral infection has rarely been addressed.

In this study, we compared the abundance of viruses and bacteria between the LRTI and non-LRTI groups but did not observe significant differences between the groups. This finding is consistent with a previous study (Man et al., 2019) that highlighted the limitations of relying solely on single microbial indicators to explain complex disease states such as LRTI. In addition, several other studies (Langelier et al., 2018; Mick et al., 2023) have developed integrated diagnostic models that combine host immune features with multi-omics microbial data, which significantly improved disease identification accuracy. Collectively, these findings suggest that a multidimensional integrative approach may be more effective in capturing biologically meaningful signals relevant to disease pathogenesis and diagnosis. Although we identified certain compositional differences in the virome between LRTI with RF and without RF, none of the viruses detected were known to be direct causative pathogens of LRTI or RF. The relatively low viral loads and absence of hallmark viral pathogens suggest that these viruses are unlikely to play a primary etiological role. Instead, the observed virome shifts are more plausibly interpreted as secondary phenomena—potentially reflecting host immune dysregulation, altered ecological niches, or microbial community restructuring in response to lung injury or infection. For instance, under conditions of pulmonary stress, whether due to infection or dysfunction, the respiratory viral community tends to exhibit reduced diversity or taxonomic shifts, indicating a disturbance in the virome equilibrium. Therefore, the virome differences between groups may reflect systemic or local host responses rather than direct viral involvement in disease pathogenesis. Accordingly, our results support this perspective: the abundance differences of individual pathogens are unlikely to fully capture the microbiological and pathological landscape of LRTI. Future research should endeavor to incorporate microbial diversity, host responses, and other clinical parameters into multidimensional analyses. Such integrative approaches may more effectively reveal biologically meaningful signals related to disease, thereby advancing LRTI diagnosis and mechanistic understanding. Our study also has several limitations. Firstly, the limited sample size and significant disparities in data volume among subgroups may introduce potential biases in the results. In particular, subgroup analyses—such as those involving LRTIs with RF—are based on very small sample sizes and may lack sufficient statistical power to support definitive conclusions. Moreover, for viral taxa with low prevalence, the ability to detect significant associations is inherently limited. However, the observed subgroup-specific trends may still suggest biologically relevant patterns that warrant further validation in larger, independent cohorts. Secondly, the absence of significant changes in bacterial community characteristics among LRTI cases, including those likely caused by bacterial pathogens, requires further investigation. In addition, although underlying diseases such as Interstitial lung disease (ILD), malignancy, or autoimmune conditions may influence the microbiome, similar comorbidities were present in both groups. Thus, the observed patterns are more likely related to infection status. Future studies with larger, stratified cohorts and improved detection methods are needed to validate these findings.

## Data Availability

The data presented in this study are deposited in the NCBI Sequence Read Archive (SRA) under BioProject accession number PRJNA1228250 (https://www.ncbi.nlm.nih.gov/sra/).
