# Supplementary material for: Metagenomic insights into respiratory viral signatures in lower respiratory tract infections with and without respiratory failure
Source: Front Cell Infect Microbiol. 2025 Sep 22;15:1637352. doi: 10.3389/fcimb.2025.1637352 (PMC12497718; doi:10.3389/fcimb.2025.1637352)
Supplement: Supplementary file 1 [file DataSheet1.pdf]

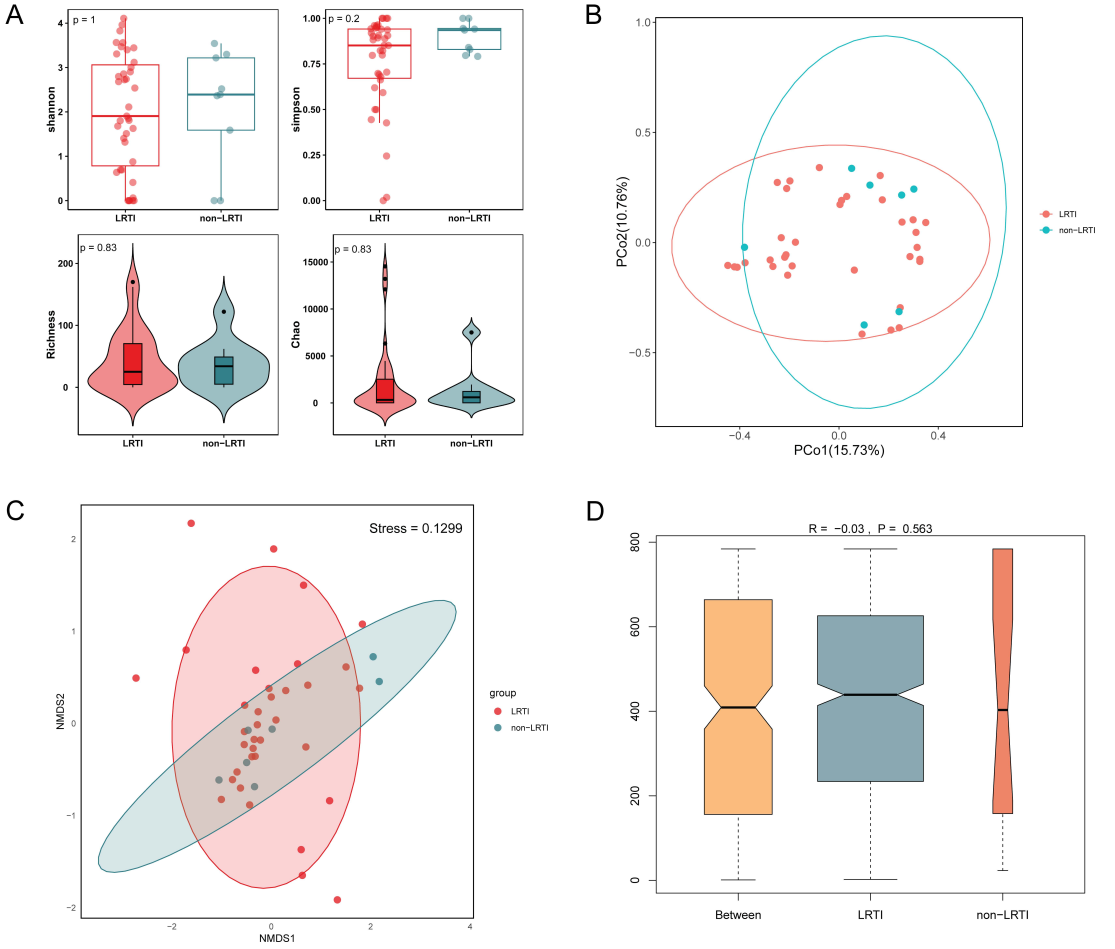

Supplementary Figure 1. Comparison of respiratory bacterial microbiota between LRTI and non-LRTI groups. (A)Alpha diversity of each group. (B)Principal Coordinate Analysis (PCoA) with Bray-Curtis distance and Classical Multidimensional Scaling (CMDS) between two groups. (C)Non-Metric Multidimensional Scaling (NMDS) analysis between two groups. (D)Analysis of Similarities (ANOSIM) test.

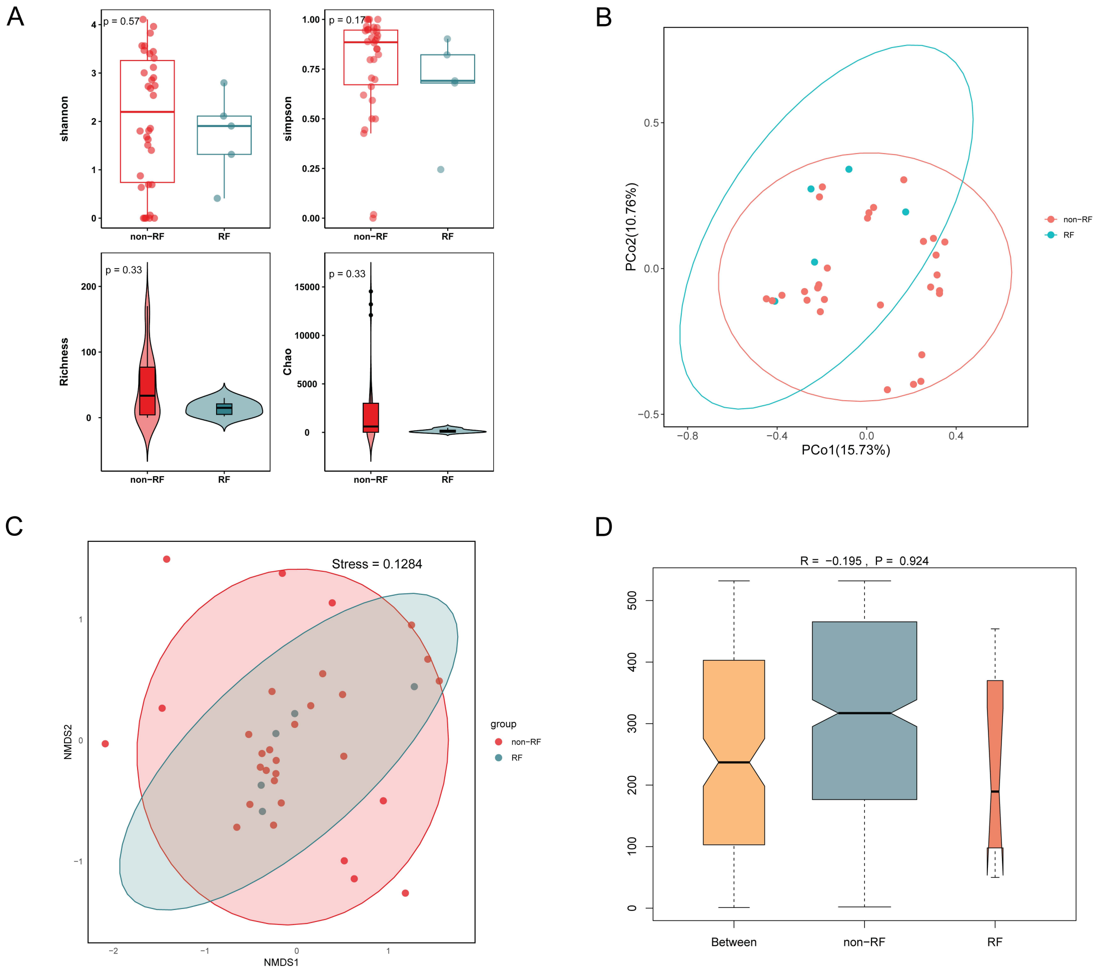

Supplementary Figure 2. Comparison of respiratory bacterial microbiota in LRTI with RF and without RF groups. (A)Alpha diversity of each group. (B)Principal Coordinate Analysis (PCoA) with Bray-Curtis distance and Classical Multidimensional Scaling (CMDS) between two groups. (C)Non-Metric Multidimensional Scaling (NMDS) analysis between two groups. (D)Analysis of Similarities (ANOSIM) test.
